# A call for change: addressing the implementation strategy using pre exposure Prophylaxis for combating the escalating HIV crisis in Zanzibar’s key populations

**DOI:** 10.1186/s12981-024-00597-5

**Published:** 2024-02-09

**Authors:** Mansour Maulid Mshenga, Weiming Tang

**Affiliations:** 1 Zanzibar Integrated HIV, Hepatitis, TB and Leprosy Programme, Zanzibar, Tanzania; 2 School of Public Health, Southern Medical University, Guangzhou, Guangndong China

**Keywords:** HIV, Key populations, Pre exposure Prophylaxis, Zanzibar

## Abstract

The Integrated Bio-Behavioural Surveillance Survey of 2022–2023 among key populations report from Zanzibar has been released. The prevalence of HIV is estimated to be 21.1%, 11.4%, and 9.3% among Female Sex Workers, Men who have Sex with Men, and People Who Injected Drugs, respectively. This has prompted a closer examination of the factors contributing to this trend, with a particular focus on the low coverage of Pre-Exposure Prophylaxis in these key populations. The current prevalence reported in December 2023 signals a critical turning point that necessitates a reevaluation of the barriers and facilitators of Pre Exposure Prophylaxis intervention to combat the epidemic. It is imperative to acknowledge the severity of the situation and take decisive action to prevent further spread of the virus in the Isles.

## Introduction

In Zanzibar, the HIV epidemic has stayed low (below 1%) in the general population since 1986, when the first three HIV clients were diagnosed but Zanzibar is known for its concentrated HIV epidemic, which is most noticeable in Female Sex Workers (FSWs), Men who have Sex with Men (MSM), and People Who Inject Drugs (PWIDs) [1].

Zanzibar offers routine HIV services, which include key interventions such as HIV Testing and Counselling (HTC), Prevention of Mother-to-Child Transmission of HIV (PMTCT), Key Populations Service (KPS), and Care and Treatment Centres (CTC) through the Zanzibar Integrated HIV, Hepatitis, TB, and Leprosy Programme (ZIHHTLP). The programme is run by the Ministry of Health Zanzibar’s Directorate of Preventive Services (DPS) and Health Education [2]. The dynamics of HIV transmission in Zanzibar are influenced by key populations because of the Isle’s concentrated prevalence of HIV. Key populations are crucial contributors in achieving a successful epidemic response in a particular background information. They also are essential partners in an effective response to the epidemic. There is proof that the sexual networks of KPs and the general public overlap [3]. This suggests that HIV is not unique among key populations, and insufficient care might undermine continued national responses as the HIV epidemic among key populations continues to persist and contribute to a significant proportion of new infections in Africa. As of 2021, these populations accounted for 51% of new HIV infections in sub-Saharan Africa, which is the most affected region of the world [4].

## Prevalence of HIV among KP in current IBBSS report of 2022–2023

Despite of the efforts to fight against HIV epidemic, the findings of current prevalence among Key Populations remains a significant challenge in Zanzibar. According to the current report of Integrated Bio-Behavioral Surveillance Survey (IBBSS) conducted in 2022/23, HIV prevalence estimated to be 21.1%, 11.4% and 9.3% among FSWs, MSM and PWID respectively [5]. These estimates are significantly higher than those from the previous IBBSS report of 2018–2019 whereby the HIV prevalence estimated to be 12.1%, 5.1% and 5.0% among FSWs, PWID and MSM respectively [6].

## Pre exposure Prophylaxis intervention in Zanzibar

In the effort to reduce the risk of HIV transmissions in December 2021, the Ministry of Health Zanzibar, through the Zanzibar Integrated HIV, Hepatitis, TB and Leprosy Programme, officially started PrEP implementation among key populations, with an estimation of 3,300 PWID, 5554 FSWs, and 2,600 MSM. By the end of December 2022, there were 1281 key populations on PrEP [7].

The lower enrolment of KPs on PrEP compared to eligible KPs estimated for this intervention marked a lower coverage, which substantially contributed to their acquisition of HIV infection besides using other biomedical interventions that already existed in the islands [8].

Based on the IBBS findings on HIV prevalence in the KPs of Zanzibar, the escalating HIV crisis in Zanzibar’s key populations underscores the newly established intervention of oral PrEP using Truvada as a priority intervention for prevention and control of HIV acquisition for key populations. The PrEP intervention introduced as pilot at Mnazi mmoja, Mwembeladu, ZAYEDESA and Chake Chake CTCs on December 2021 by Ministry of Health Zanzibar through Zanzibar Integrated HIV, Hepatitis, TB and Leprosy Programme (ZIHHTLP) initiated and supported by Center for Disease Control (CDC) of United States of America [7].

Pre Exposure Prophylaxis is the use of antiretroviral drugs (ARVs) by people who do not have Human Immunodeficiency Virus infection in order to prevent the acquisition of HIV [9]. It offers option of prevention to high exposure groups at high risk of acquiring and transmitting HIV infection which include key populations [10].

PrEP is highly effective for preventing HIV infection when taken as prescribed, studies have confirmed that PrEP reduces the risk of acquiring HIV from sex by about 99% while from injection drug use by at least 74% [11].

The World Health Organization (WHO) recommends that oral PrEP containing Tenofovir Disoproxil Fumarate (TDF) or Tenofovir Alafenamide (TAF) in combination with Emtricitabine (Truvada) should be offered as an additional prevention choice for people at substantial risk of HIV infection as part of combination HIV prevention approaches [12].

The establishment of Pre-Exposure Prophylaxis intervention in Zanzibar is to lower the risk of HIV infection and prevent the virus from spreading to key populations by providing medication before an individual is exposed to the virus. This approach is part of a larger strategy to stop the HIV pandemic in Zanzibar [7].

Despite of the concerted effort of two years implementation of PrEP interventions, HIV epidemic continues to impact key populations in Zanzibar evidenced by report introduced on December, 2023 of Tanzania HIV Impact Survey and Integrated Bio Behavioral Surveillance Survey of Zanzibar 2022/2023 [5].

The success of the intervention that was implemented in the Isles to target the key populations is a matter of increasing concern in the wake of this report. This suggests that Pre Exposure Prophylaxis efforts in the Isles are limited in their effectiveness by enduring obstacles, including cultural stigmas and insufficient healthcare support 3].

## Recommendation

The connection between low PrEP coverage and the rising prevalence of HIV in Zanzibar’s key populations underscores the urgent need for comprehensive interventions. By addressing the barriers to PrEP access and uptake, the region can make significant strides in curbing the spread of HIV and safeguarding the health and well-being of its key populations. Qualitative implementation research for Pre Exposure Prophylaxis implementation is crucial for identifying barriers and facilitators in order to pave the way for feasible approaches that can truly lower the prevalence of HIV in Zanzibar, as the intervention has been demonstrated to be successful when carried out consistently as recommended.

## Conclusion

Considering the high prevalence of HIV among key populations in Zanzibar, the Ministry of Health Zanzibar, along with the Zanzibar Integrated HIV, Hepatitis, TB, and Leprosy Programme, should take the necessary steps as recommended for implementing PrEP and investigate viable options to reach high coverage for its appropriate and effective implementation in order to prevent the spread of HIV, particularly among KPs in the Isles.

## Data Availability

No datasets were generated or analysed during the current study.
